# Facilitators and barriers to physical activity in patients in an acute care hospital setting from an interprofessional perspective: A qualitative study

**DOI:** 10.1177/02692155251325614

**Published:** 2025-03-11

**Authors:** Gesche Gertz, Gesche Ketels, Susanne GR Klotz

**Affiliations:** Department of Physiotherapy, 37734 University Medical Center Hamburg-Eppendorf, Hamburg, Germany

**Keywords:** Physical activity, health literacy, hospital, influencing factors, qualitative research

## Abstract

**Objective:**

Physical activity levels of patients in hospital are low. The aim of this study was to explore the perceptions of physiotherapists, nurses, and physicians about physical activity of hospitalized patients.

**Design:**

Qualitative focus group study using phenomenology.

**Setting:**

University hospital.

**Participants:**

Thirty healthcare professionals (physiotherapists, nurses, and physicians) participated in six focus groups.

**Main measures:**

Semi-structured focus groups following an interview guide were conducted. Besides identification of influencing factors and development of ideas for associated interventions, participants should reflect on the roles of different professions. Content analysis of the transcriptions was performed in iterative cycles involving three researchers using inductive coding and redefining based on the literature. Consensus was reached through discussions in the research team.

**Results:**

Three themes of influencing factors on physical activity of patients in hospital with seven codes emerged. In the theme “patient” physical and psychosocial factors were mentioned. The theme “organization” pooled all factors regarding facilities and processes. The third theme “health literacy” has an impact on the two other themes. In this theme self-efficacy, handling of health literacy/knowledge, and communication and cooperation became apparent as codes.

**Conclusions:**

Health literacy plays an important role in the physical activity of patients in hospital. The developed model of influencing factors on physical activity highlights the importance of health literacy for all stakeholders, including patients, staff, and the organization and illustrates the connections to other influencing factors. This model can be helpful in conceptualizing interventions to promote physical activity.

## Introduction

Physical activity has positive effects on physical, cognitive, and psychosocial dimensions regardless of the setting.^1,2^ The World Health Organization defines physical activity as “any bodily movement produced by skeletal muscles that requires energy expenditure.”^2^^(p. 1452)^ According to the World Health Organization’s 2020 guidelines, adults with or without chronic conditions should undertake regular physical activity of at least 150–300 minutes of moderate or at least 75–150 minutes of vigorous-intensity aerobic physical activity. However, even if these recommendations could not be met, doing some physical activity is better than doing none.^2^

Patients, who stay physically active during hospitalization, benefit in physical, psychological, social, and organizational outcomes.^3^ However, patients in hospitals achieve insufficient levels of physical activity and spend 87–100% of a 24-hour time interval lying in bed or sitting. This is valid for patients across all ages and different medical specialties.^4^ The hospital setting should be used to motivate patients to be more active and to pave the way toward a sustained lifestyle change.^5,6^ Patients name uncertainty about physical symptoms, their own and the staffs’ motivation, lack of knowledge about physical activity and lifestyle change, as well as structural and logistical barriers as influencing factors.^7,8^ One strategy to improve physical activity in patients in hospital might be increasing the health literacy. Lower levels of health literacy are associated with lower levels of physical activity^9,10^ and with lower levels of motivational readiness for leisure-time physical activity. Nonetheless, health literacy was often not included in interventions aiming at the promotion of physical activity in patients in hospital.^11^

The promotion of physical activity is regarded as an interprofessional task for different healthcare professionals; however, most of the existing studies about perceptions of healthcare staff have interviewed nurses alone.^7,12^ Nonetheless, promotion of physical activity in the hospital setting should be acknowledged as an interprofessional approach.^13^ Therefore, the study aimed to explore the perceptions of physiotherapists, nurses, and physicians about physical activity in patients in hospital and the facilitating factors and barriers to physical activity.

## Methods

To understand the factors influencing physical activity in a hospital environment from the perspective of healthcare professionals, a qualitative exploratory design with phenomenology using focus groups and qualitative content analysis with an interpretative nature^14^ was selected. The study took place in November 2018 and February 2020 at the University Medical Center Hamburg-Eppendorf in Hamburg, Germany. The University Medical Center Hamburg-Eppendorf is an urban tertiary care academic hospital with approximately 1800 beds. Ethical approval was given by the Institutional Review Board of the German Association of Physiotherapists (reference number 2019-08), and the study was registered with the German Clinical Trials Register (reference number DRKS00019810). Reporting of this study is in line with the COREQ checklist.^15^

### Participants

Physiotherapists, nurses, and physicians were recruited using newsletters, emails, presentations, and personal contacts. They were eligible if they were employees of the University Medical Center Hamburg-Eppendorf, had either professional recognition or were at least second-year students in one of the professions, and had sufficient German language skills. All participants signed written informed consent.

### Data collection

Prior to data collection, the researchers have gathered their assumptions (Table 1). Semi-structured focus groups within each profession, striving for a group size of five to eight participants^16^ were conducted. As a socially oriented method, focus groups offer the advantage of providing data from a social structure flexibly and quickly. In a protected space and through group dynamics, they can bring out aspects that would possibly not have developed in individual interviews.^17^ The focus groups were structured using a pilot-tested interview guide (Table 1) and consisted of three parts: identification and grouping of influencing factors on physical activity in hospitals, development of targeted interventions supporting or preventing the factors previously elaborated, and discussion of the roles and responsibilities of different healthcare professionals in patients’ physical activity. The group conversation was accompanied by designing a metaplan clustering factors and corresponding interventions. The focus groups took place in a conference room and were led by the last author (SGRK: PhD, female senior researcher with experience in qualitative research and a trained physiotherapist). A second person from the research team was present assisting the moderator and taking field notes. Both persons introduced themselves with their profession and their role in the project. The focus groups were audiotaped, and pictures of the resulting metaplan were taken.

**Table 1.** Focus group structure guide with assumptions of the researcher, instructions, and interview questions.

Assumptions of the researcher about the topic/focus groups: physical activity of patients in hospitals is influenced by many related factors including behaviour and circumstances; physical activity is a task for all healthcare professionals and not only for physiotherapists; the promotion of physical activity requires a cultural change in the hospital.
(1) Welcome and introduction Short overview of the content of the focus group, clarification of organizational questions, round of introductions of the participants.
(2) Individual brainstorming on facilitating and hindering factors Approximately 5 minutes brainstorming phase for the participants to write down their thoughts about possible influencing factors of physical activity on green and red cards. “Please write down on the green and red cards factors you perceive as promoting or impeding for physical activity in patients in hospital.”
(3) Discussion of facilitating and hindering factors in plenary Presentation of the cards, joint arrangement of the cards and discussion. “Group your cards into cluster. Is there anything else missing? Would you group the cards differently? Why?”
(4) Brainstorming in small groups of two or three participants on possible interventions and strategies Approximately 10 minutes brainstorming in small groups about possible interventions and writing the ideas on blue cards. “Please exchange ideas of interventions with targeting either facilitating or hindering factors with your partner and write them down on the cards.”
(5) Discussion of interventions and strategies in plenary Presentation of the cards, arranging the cards together with the cards already on the table and discussion. “Group your cards into the existing cluster. Is there anything else missing? Can you think of any other interventions? Would you group the cards differently? Do the intervention cards lie with the appropriate factors or do the interventions address additional or different factors? Why?”
(6) Mapping of roles and responsibilities for enhancing physical activity Marking the perceived responsibilities of the individual professions for the various proposed interventions with the help of different stickers. “Please consider which of the interventions and measures on the cards are the tasks of the respective professional groups and mark them with stickers.”
(7) Discussion in plenary Discussion about the allocated intra- and interprofessional responsibilities. “Why do you think the task with the sticker are the task of physiotherapy/nursing/medical profession/common task of all professional groups? What qualifies the professional group especially for this task?”
(8) Conclusion and farewell Summary of the focus group session, outlook for the next steps in the project, and acknowledgment for the participants.

Annotation: The instructions and questions were translated into English.

### Data analysis

Digital audio recordings were transcribed verbatim^18^ and de-identified. The transcripts were analyzed using different colored pens and paper using an iterative team-based approach to coding.^19^ In the first cycle, transcripts were read to get a sense of the whole. Afterwards, they were reviewed and first themes and codes were created inductively along the material. These themes and codes were collected in a codebook. To minimize the impact of a preconceived mindset, these first steps were done by first and last authors (GG, SGRK) independently and a third researcher (GK), previously not involved in the focus group conduction, was involved in the analysis. In the next step, the research team compared, discussed, and reached consensus about the themes and codes as well as about the codebook. Reflections regarding themes were noted and discussed. The emerging themes were grouped and related in a model, which was compared to literature and redefined. Thereafter, the researchers reviewed and coded the material once more independently based on the emerging model. In the last step, the final model was discussed and consented to. The German quotes from participants were translated into English by the authors and proofread by a native speaker.

## Results

All persons who responded to the invitation also participated in the study. Two focus groups per profession with four to seven participants were realized lasting between 74 and 127 minutes (mean 107 minutes). Thirty healthcare professionals (23 women, 76.7%) participated; 11 of them were nurses (82% female), 8 were physicians (50% female), and 11 were physiotherapists (91% female). Their professional experience ranged from second-year students to 30 years of experience with a mean of 18 years. Details about each participant can be found in Table 2.

**Table 2.** Details of the participants.

Profession	Participant number	Gender	Years of work experience	Main area(s) of work
Physiotherapist	01	f	28	Outpatient clinic
	02	f	7	Cardiology, outpatient clinic
	03	f	7	Intensive care unit
	04	f	19	Neurology
	05	f	19	Outpatient clinic
	06	f	30	General surgery
	07	f	20	Gynecology and obstetrics
	08	m	20	Internal medicine, orthopedics
	09	f	36	Lecturer in the physiotherapy degree program
	10	f	0	Third year in the physiotherapy degree program
	11	f	0	Third year in the physiotherapy degree program
Nurse	12	f	35	Dermatology
	13	f	43	Dermatology
	14	f	7	Infectiology
	15	f	20	Infectiology
	16	f	20	Infectiology
	17	f	22	Dermatology
	18	m	10	Dermatology
	19	m	15	Intensive care unit
	20	f	30	General, visceral, and thoracic surgery
	21	f	2	General, visceral, and thoracic surgery
	22	f	5	General surgery
Physician	23	m	18	Orthopedics
	24	m	14	Visceral surgery
	25	m	10	Visceral surgery
	26	m	10	Vascular surgery
	27	f	4	Neurology
	28	f	14	Urology
	29	f	20	Oncology
	30	f	10	Gerontoanesthesia

f = female, m = male.

**Figure 1.**
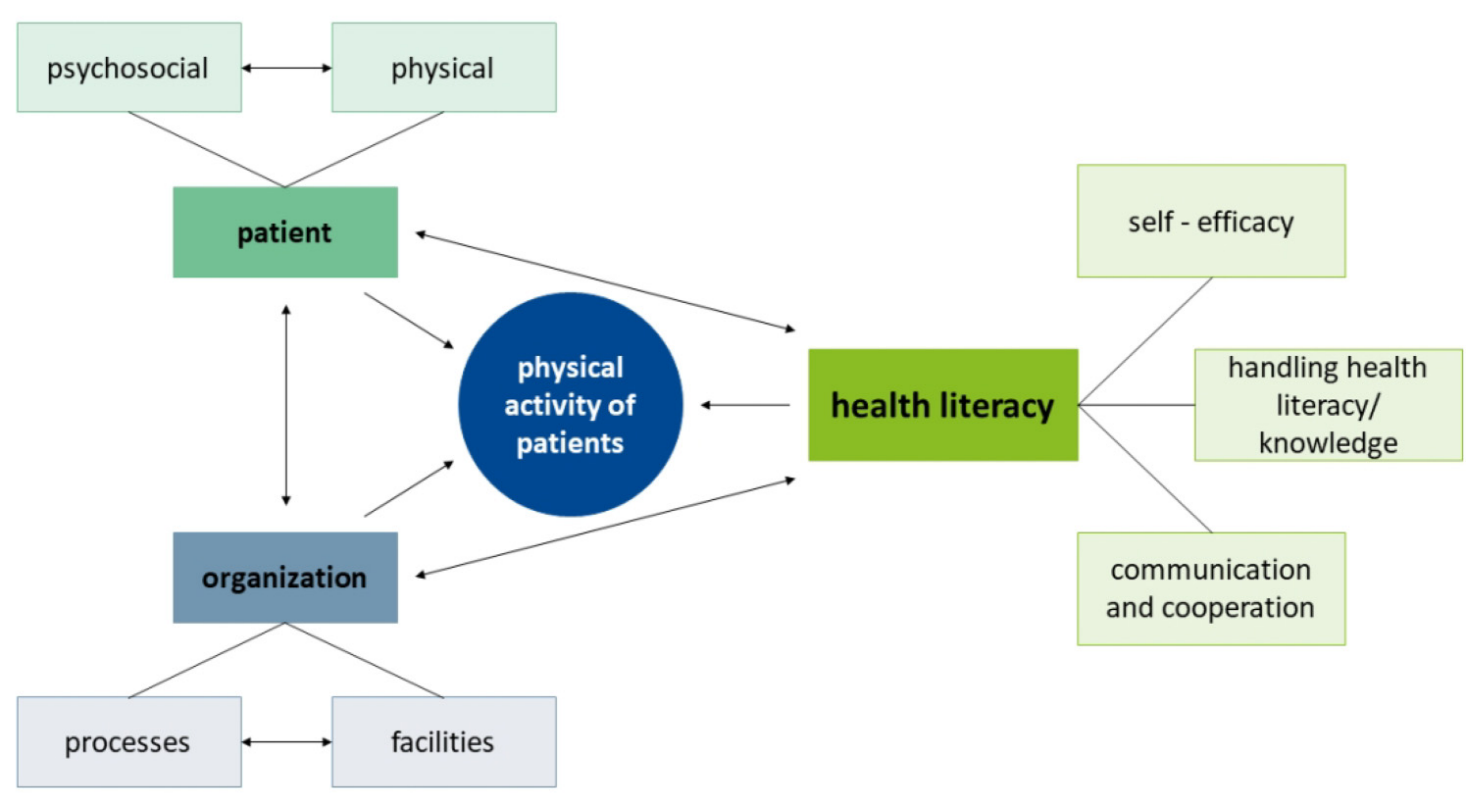
Model of factors influencing physical activity of patients in hospital.

Three themes with seven codes emerged. Figure 1 shows the connections between the themes in a model describing the influencing factors of physical activity of patients in hospital.

### Patient

The state of the patient either physical or psychosocial was one theme. Strength was regarded as important for mobilization and physical activity, both the higher prehospital strength and the acute loss of strength during hospitalization. This could be attributed to different factors, which are connected resulting in a downward spiral. Influencing factors include the general condition, nutritional status, and appetite.

In addition, consequences and limitations due to the current health problem are subsumed in this theme, for example, fatigue, reduced vigilance, or prescribed bed rest. Delirium was recognized as a complex problem. Participants saw the importance of being physically active as prevention of developing delirium. However, they recognized the negative consequences for patients with acute delirium and who might be physically restrained. In connection with the physical state, participants named medical necessities like intravenous lines, probes, and catheters as influencing factors. Patients can experience discomfort or pain and feel uncertain if they can or are allowed to move with medical devices.

[…] the accesses, catheters, drains, […], everything that somehow ties the patient to the bed […], but for the patient alone it is then difficult to say “I'm getting out of bed now” because he is attached to fifteen tubes. [05]

One of the important factors was pain influencing the patient both physically and psychologically and leading to avoidance of physical activity.

But someone, who is in so much pain, despite the best education, will say “that's all well and good, I know that too, but I'm not moving here anyway, because it hurts me like a pig”. [02]

Some participants believed that the bigger problem was not pain itself, but the fear of pain. Pain management was regarded as an interprofessional task with pain medications being given more liberally to decrease the fear of movement and facilitate physical activity. Additionally, pain levels should be closely monitored with adequate instruments like the visual analog scale or the numeric rating scale.

Altogether, fear was a common subject with pain-related fear, fear that operation wounds could tear, fear of getting up, fear of medical devices, or fear that something negative could happen.

Among the affective factors, motivation was highlighted. Intrinsic motivation to be physically active could be drawn from different motives, for example, movement itself and the fun associated with it, or movement as a prerequisite for experiencing something. One controversial example of intrinsic motivation discussed was the effect of craving in patients, who smoke. Often, they mobilize themselves more quickly to smoke outside.

On the other hand, if patients had low levels of motivation, they might be more physically inactive and let the personnel take action for them.

Finally I can lie here and do nothing and others take care of me. [09]

However, some participants stressed the self-responsibility of patients being active.

[…] but he's not a child, he's an adult, he practically has to take responsibility for himself. [02]

### Organization

The second theme was the organization with its structures, processes, and staff members. A corporate culture embedding physical activity as a central value has a high impact on strategic decisions and physical activity would be acknowledged in planning and execution. Role models might be helpful as multipliers, as they can raise awareness, provide information, and exemplify physical activity.

I think good role models would be great. If there are people in the teams, who do not just write movement is important on a piece of paper, but people, […] who lead the way. [19]

Structures and processes in hospitals are often designed with the bed as the centerpiece, for instance, served meals, urine bottles at the bedside, and patient transport devices. Furthermore, patients often wear nightclothes during the day spending most of their time in bed. This goes hand in hand with passivity and assuming a supposed patient role.

The organization with its physical environment could be a facilitator for physical activity by having stimulating, meaningful, and attractive effects.

[…] rooms also have to invite movement, so if it's such a junk room and so totally cramped, blocked with food trolleys […], and then as dark as possible, walls are [having] nothing on it, then that's not inviting to movement. [09]

Several opportunities were named covering smaller modifications like start and stop lines on the floor or leaflets with walking routes but also bigger projects like a tea kitchen on the ward or a park on the campus.

Disturbances in patients’ rooms caused by persons entering the room for visiting rounds, examinations, treatments, cleaning, and housing were perceived as not supportive. In addition, if patients leave their room and walk down the ward corridor they might get the impression that they disturb the ward routine.

Organizational and interprofessional processes should be designed to promote physical activity in patients. This includes, for instance, the temporary or early removal of medical equipment that ties patients to their beds. In addition, organizational processes should focus on the promotion of dressing in day clothes and getting up instead of remaining in the hospital gown in bed.

The people can't get out of bed when the [dialysis] is running, […] and it […] runs for hours a day, you have to coordinate with […] the care and if it does not fit, then it is stupid for the patient, then he sometimes stays the whole day in bed. [05]

One subject was the lack of time as a hindering factor, often attributed to staff shortages, but also to the processes on the ward. Lack of time might contribute to a lower level of physical activity, as patients were uncertain about moving without assistance.

Better interprofessional cooperation optimizing patient outcomes and increasing satisfaction was demanded. Communication including the formal information about patients during rounds, but also the informal talks between healthcare professionals, is needed. In contrast to nurses, who often take care of only a limited number of patients, physiotherapists are often responsible for the entire ward. Hence, their contact with individual patients is limited stressing the need for interprofessional communication. Participants also suggest case conferences with physical activity as a central subject.

The different healthcare professionals should coordinate their tasks in favor of patients’ physical activity level. However, participants stressed their needs to be seen and respected as equal partners. Although the first few mobilizations and other physical activity units were often regarded as the expertise of physiotherapy, the physical activity of patients was recognized as the responsibility of all healthcare professionals.

Participants pointed out that currently preoperative interventions are neglected. If patients were provided with information in advance, they could prepare themselves cognitively and mentally for the upcoming hospital stay in relation to expectations about physical activity.

[…] however [the patients] come in, […] with slippers. Of course we provide them with some hotel shoes or with anti-slip socks, but that they are told preoperatively “Watch out, you need this and this to get back on your feet, footwear” […]. [08]

In addition, if patients could preoperatively train postoperative behavior, for instance being able to ambulate with crutches, they would be more active and their fear might be reduced. Preoperative interventions could also be helpful for healthcare professionals when they get to know the patients, their recent levels of physical activity, and their individual goals.

### Health literacy

Health literacy was the topic underlying all other issues. It was defined in accordance with the definition from the Healthy People 2030 initiative^20^ as the ability to find, access, understand, appraise, and use information or services for health-related decisions and actions. Health literacy was regarded as important for the effective self-management of patients. Education was regarded as a possibility to improve health literacy. Moreover, health literacy had the potential to decrease uncertainty about physical activity while empowering patients.

Patients with sufficient health literacy can take responsibility for their physical activity. They can set their own goals and manage themselves. Supporting materials, like diaries or calendars, were considered helpful in increasing knowledge and motivation. Furthermore, the level of health literacy shapes the attitudes and beliefs of patients about the hospital.

they have an image of the hospital as a reclining ward, […], that they think “In the hospital, that's where I lie down, that's where they take care of me and make me healthy”, in other words, a passive kind of hospital. [09]

This goes hand in hand with passivity and assuming a supposed patient role.

the patients, when they walk through the entrance here in front actually give up everything including personality, but also clothing-wise and get into their pyjamas and get into bed. [13]

Health literacy to increase physical activity was not only relevant for the patients but also for their loved ones. They could take patients for a walk or encourage them to eat, which would support and relieve the healthcare professionals. Moreover, to increase their health literacy, loved ones could also be included in education sessions together with the patients.

There are also relatives […], who tend to stop the movement of the husband or whoever, “Oh, God, but don't do that”, and then there are those, who are themselves somehow totally inhibited and then there are those, who are there every day and also do the exercises. [03]

For healthcare professionals and all personnel working in the hospital, health literacy influences their behavior in terms of interaction with the patients, interaction with other staff members, and the design of the hospital. Health literacy was seen as a prerequisite to interprofessional communication and cooperation and was connected to motivation.

[My colleagues] just dismiss it a bit and say: “Oh, that's not really that important” […] They are also not sufficiently informed about the positive effects of physical activity. [28]

## Discussion

This qualitative study explored facilitators and barriers to physical activity for patients in an acute care setting from an interprofessional point of view using six focus group discussions with 30 healthcare professionals. The three themes “health literacy,” “patient,” and “organization” emerged resulting in a model of influencing factors for physical activity in hospital settings with health literacy as the key element.

According to the participants, several physical factors influence physical activity including medical necessities. This is in line with previous findings such as patients spending significantly more time in bed when being attached to an intravenous line or having a urinary catheter.^21^ Early removal of probes and catheters, when possible and medically justifiable, can increase early ambulation rates and the level of physical activity.^22^ Pain experienced by the patients was regarded as one of the most important barriers by the participants. Patients might feel that they cannot be physically active due to the pain.^23^ These attitudes were also present in healthcare professionals influencing the interaction with patients.^23^ Furthermore, fear of pain influences the level of pain intensity, the consumption of analgesics, and the experienced comfort level.^24,25^ Several studies could demonstrate a decrease in pain in patients when being physically active instead of an increase.^3^ Hence, pain and pain of fear might be worth addressing in multimodal programs to promote physical activity.^21,23^

Some of the participants mentioned the influence of wearing nightclothes during the daytime. Patients might be perceived as being sick and dependent on help and instructions and they might adopt a passive, immature patient role while wearing gowns or nightwear. This phenomenon is described as pyjama paralysis.^26–28^ Participants of the focus groups stressed the need for changing the picture of patients in the hospital for patients but also for healthcare professionals. They acknowledged their own role in the cultural change needed in hospitals to improve physical activity.^29^ Multimodal interventions have been demonstrated to be effective in terms of patient dress, mobilization, and physical activity.^30^ In addition, participants concluded that all healthcare professionals are responsible for the hospital mobility of patients. However, the different professions have different expertise and their specific competences and tasks should be made clear within the team.^29^

Moreover, participants named the hospital organization with its structures and processes as a contributing factor to physical activity. A culture of bed rest prevails with the bed as the centerpiece and only few incentives to leave the bed or the patient room.^31^ The participants emphasized the need for an enriched environment with activities and tasks to do having incentives to get up. Furthermore, in line with the literature,^26^ they criticized the frequent disturbances in the patient room as not supportive of physical activity. Enriching the environment with stimulating resources and communal areas can result in higher activity levels and more social activities among patients.^32^ With a sophisticated hospital design, patients and health staff could benefit alike.^33^

The image and concept of hospitals as well as the behavior of persons in hospitals are constructed socioculturally.^34^ Thus, behavior is a reflection of health literacy in the society but also of the individual.^35^ If a person, either a patient or healthcare professional, has a concept of hospitals with passive patients, this image will influence their behavior towards physical activity.^36^ It became apparent during the focus groups that words and behavior mirror health literacy. Conflicting messages from different healthcare professionals regarding the allowance of physical activity, the amount of bed rest, and possible risks when being physically active could upset patients and result in fear-avoidance behavior. Perceived lack of time was often expressed, especially by nurses, which might result in the prioritization of medical tasks over engaging in physical activity. However, the prioritization of medical tasks while neglecting physical activity could also be due to a lack of knowledge.^37^

The resulting model emphasizes the central role of health literacy and its impact on patients and the organization with personnel, structures, and processes. Former studies have demonstrated that persons with high levels of health literacy are more physically active.^9,10,37^ Nevertheless, nearly half of the European general population reported to have insufficient health literacy.^38^ Among healthcare professionals, one-fourth were reported to have insufficient health literacy, which is associated with a lower usage of communication techniques.^39^ Thus, the improvement of health literacy of patients can be addressed when improving the health literacy of healthcare professionals.^40^ Using their improved competencies, healthcare professionals can improve the health literacy of patients with tailored educational interventions addressing different themes, including those mentioned in the focus groups like pain and fear of pain.^41^ A higher level of health literacy can improve the self-management of patients^42^ and is significantly correlated with behavior change leading to more physical activity.^42,43^ In addition, the creation of a common understanding of hospital health literacy of all staff members, including administration, could be beneficial in the cultural change towards higher physical activity of patients in hospitals.^23,29,44^

The study has some limitations. First, participants were recruited from a single hospital. In addition, participants were recruited, among other strategies, via personal contacts, which might result in selection bias.^45^ However, different passive and active sampling strategies were combined into a multi-modal approach for recruitment putting the potential selection bias into perspective. These multi-modal recruitment approaches were advocated to facilitate the recruitment of healthcare professionals.^46^ Second, the focus groups were conducted within each profession instead of mixing professions. Mixed focus groups might make a valuable contribution to the development of knowledge about a specific topic, as the participants can reflect on their different professional insights.^47^ Nonetheless, our study is one of only few including the three professions physiotherapists, nurses, and physicians.^12^ These professions are frequently perceived as those being responsible for the topic of physical activity of patients with physiotherapists commonly described as the most appropriate ones.^48^ Nevertheless, future studies might look at other healthcare professions and their perspective on this topic. This study resulted in a theoretical model of influencing factors focusing on the impact of health literacy, a topic neglected in acute care settings.^49^ However, future studies might explore if the model can be also suitable in other settings.

In conclusion, the facilitators and barriers to physical activity according to this study can be summarized in the domains of health literacy, the patient, and the organization, while highlighting the importance of health literacy for all stakeholders. One element of effective multimodal interventions promoting physical activity in hospitals should be addressing the health literacy of patients, healthcare professionals, and organizations.

**Clinical messages**

- The physical activity of patients in hospitals is influenced by a variety of topics covering patient and organizational factors and is regarded as a multiprofessional responsibility.
- Enhancing the health literacy of patients, healthcare professionals, and healthcare organizations may result in an increase in physical activity among patients during their hospitalization.
- Sustainable improvement in the physical activity of patients in hospitals requires a cultural change towards a culture of physical activity.
